# Optimized Microbial Scaffolds Immobilized with *Pleurotus ostreatus* and *Aspergillus oryzae* on Foaming Bacterial Cellulose

**DOI:** 10.3390/ma18133151

**Published:** 2025-07-03

**Authors:** Pei-Ching Chan, Wei-Lun Ku, Yung-Kun Chuang, Yu-Chieh Chou, Chen-Che Hsieh, Yung-Kai Lin, Shella Permatasari Santoso, Shin-Ping Lin

**Affiliations:** 1 School of Food Safety, Taipei Medical University, 250 Wu-Hsing Street, Taipei 11031, Taiwan; 2 Ph.D. Program in Drug Discovery and Development Industry, Taipei Medical University, 250 Wu-Hsing Street, Taipei 11031, Taiwan; 3 Department of Seafood Science, College of Hydrosphere, National Kaohsiung University of Science and Technology, Kaohsiung 81157, Taiwan; fstcch@nkust.edu.tw; 4 Institute of Food Safety and Risk Management, National Taiwan Ocean University, Keelung City 20224, Taiwan; yklin@mail.ntou.edu.tw; 5 Department of Chemical Engineering, Faculty of Engineering, Widya Mandala Surabaya Catholic University, Kalijudan 37, Surabaya 60114, Indonesia; shella_p5@yahoo.com; 6 Chemical Engineering Master Program, Widya Mandala Surabaya Catholic University, Kalijudan 37, Surabaya 60114, Indonesia; 7 Collaborative Research Center for Zero Waste and Sustainability, Widya Mandala Surabaya Catholic University, Kalijudan 37, Surabaya 60114, Indonesia

**Keywords:** *Pleurotus ostreatus*, *Aspergillus oryzae*, foaming bacterial cellulose, immobilization

## Abstract

In this study, we explored the development and characterization of fungus-immobilized foamed bacterial cellulose (FBC) scaffolds using *Pleurotus ostreatus* and *Aspergillus oryzae*. FBC, a porous biomaterial with high structural integrity and resistance to enzymatic degradation, served as a three-dimensional matrix for fungal cultivation. The results indicated effective fungal immobilization, with the 1% *A. oryzae*-immobilized FBC group (FBC/1A) achieving the highest production yield. The water content (97%) and swelling behavior (95.9%) analyses revealed that *P. ostreatus*-immobilized FBC maintained high hydration levels and rehydration capacities, whereas *A. oryzae* immobilization led to slightly reduced water retention. Morphological assessments via SEM confirmed the presence of fungal-derived fibers integrated with native cellulose structures, suggesting successful immobilization. A thermogravimetric analysis demonstrated enhanced thermal stability in fungus-immobilized FBC, particularly in the *A. oryzae* group, while FTIR spectra suggested possible structural alterations induced by fungal activity. Collectively, these findings support the potential of fungal-immobilized FBC as a robust, biodegradable material with promising applications in biotechnology and sustainable material development.

## 1. Introduction

Bacterial cellulose (BC) is a biopolymer produced from microorganisms [1]. The intrinsic features of BC, such as its three-dimensional (3D) nanofibrous architecture [2], make it particularly suitable for immobilizing microorganisms, including fungi [3]. The large surface area of BC promotes efficient fungal adhesion and proliferation, which are essential for various bioengineering processes, such as bioremediation, enzyme production, and biosensing. Notably, the global bacterial cellulose market is projected to reach USD 700 million by 2026, reflecting its increasing importance in industrial and biomedical applications [4]. Additionally, BC possesses high mechanical strength, which may support its long-term use as a scaffold for microbial immobilization [5]. The high water-holding ability of BC also supports a moist environment [6,7], which is crucial for fungal metabolism and activity, while its biocompatibility minimizes the risk of adverse effects on the immobilized cells [8]. Consequently, BC is increasingly recognized for its outstanding potential as an immobilization matrix [9] in biotechnological applications, due to these unique material characteristics [10].

Foaming BC (FBC) is a porous BC produced by a foam medium during BC production. This FBC-producing system involves the use of foaming agents, inert gases, or the combination of hydrogel and freeze-drying methods to produce FBC with lower densities and increased surface areas [11]. FBC retains the mechanical strength and biocompatibility [12] of traditional BC while offering excellent water absorption [13] and flexibility [14], making it highly promising for biomedical [11] and environmental materials applications [15].

*Pleurotus ostreatus*, commonly known as the oyster mushroom, is well known for its robust ligninolytic enzyme system [16], which can break down complex organic pollutants [17], making it ideal for environmental cleanup efforts. *Aspergillus oryzae* exhibits high production of multiple enzymes [18] and a strong biosorption capacity [19], making it a promising candidate for various applications, such as industrial enzyme production [20], biotransformation [21], and pollutant removal [22,23].

In this study, *Komagataeibacter xylinus* was utilized to produce FBC, while two specific fungi were immobilized on the FBC scaffolds. The material characteristics, such as the morphology, swelling behavior, functional group identification, and thermostability of fungus-immobilized FBC, were further investigated. These findings highlight the potential of fungus-integrated FBC as a multifunctional biomaterial platform for future applications in biotechnology, bioremediation, or functional packaging systems.

## 2. Materials and Methods

### 2.1. Materials

Potato dextrose broth (PDB; Neogen, MI, USA) and corn steep liquid–fructose (CSL–fructose) culture media were individually sterilized at 121 °C for 15 min. The foaming CSL–fructose medium was modified with xanthan (Danisco, Copenhagen, Denmark) and cremodan (Danisco) to serve as the foaming medium. The bacterial strain *K. xylinus* ATCC 23769 and the fungal strains *P. ostreatus* 36069 and *A. oryzae* ATCC 10124 were all sourced from the Bioresource Collection and Research Center (BCRC; Hsinchu City, Taiwan). *Komagataeibacter xylinus* was stored at −80 °C, while *P. ostreatus* and *A. oryzae* were cultivated on PDB agar under static conditions at 28 °C for 14 days and then stored at 4 °C.

### 2.2. Preparation of the Culture Strain

To prepare BC, *K. xylinus* was cultivated in CSL–fructose medium and incubated statically at 28 °C for 3 days. *Pleurotus ostreatus* and *A. oryzae* were preserved on PDA plates at 4 °C. For cultivation, a 1 cm diameter section of each strain was placed into PDB medium and incubated in a shaker (180 rpm) at 28 °C for 3 days.

### 2.3. Production of FBC

Preparation of the modified foaming medium was based on a method described by Rühs et al. in 2018 [11]. The CSL–fructose medium, supplemented with 0.2% xanthan and 1% Cremodan (along with trace elements), was used as the modified foaming medium. This medium was mixed with a 1% inoculation of *K. xylinus*, foamed for 10 min using a commercial milk frother at its highest speed, and then incubated statically at 28 °C for 10 days. The FBC produced from the CSL–fructose foaming medium was compared to FBC produced from the same medium but inoculated with different fungi.

### 2.4. Immobilization of Fungi on FBC

For FBC with the *P. ostreatus* group (FBC/P), the modified foaming medium was mixed with a 1% inoculation of *K. xylinus* and various concentrations of *P. ostreatus* (0.5%, 1%, 2%, and 4%). The mixture was then foamed for 10 min using a commercial milk frother at its highest speed and subsequently incubated statically at 28 °C for 10 days.

For FBC with the *A. oryzae* group (FBC/A), the modified foaming medium was similarly mixed with a 1% inoculation of *K. xylinus* and different concentrations of *A. oryzae* (0.5%, 1%, 2%, and 4%). The mixture was foamed for 10 min with a commercial milk frother at its highest speed and incubated under static conditions at 28 °C for 10 days.

### 2.5. Physicochemical Characterization of FBC

#### 2.5.1. Water Content and Swelling Behavior

FBC samples were soaked several times in 0.1 N NaOH at 80 °C in a water bath for 10 min. Afterward, the samples were weighed before and after freeze-drying to calculate their water content (WC). The WC was determined using the wet and dry weights of FBC, calculated with Equation (1):

Original water content (%) = ((Wt − W0)/Wt) × 100%(1)

where W0 and Wt represent the weights of dried FBC and wet FBC, respectively.

To evaluate the swelling ratio of FBC, the freeze-dried FBC was weighed and immersed into double-distilled (dd)H_2_O for 24 h to obtain the wet weight, and the swelling rate was calculated using Equation (2):

Swelling rate (%) = ((Ws − W0)/W0) × 100%(2)

where W0 represents the weight of the original freeze-dried FBC, and Ws represents the weight of swollen FBC. The same steps were repeated for the swelling BC sample to determine the reswelling rate.

#### 2.5.2. Morphological Analysis

Freeze-dried FBC samples were coated with a thin layer of gold and analyzed using a field-emission scanning electron microscope (FE-SEM; Hitachi S-4800, Hitachi, Tokyo, Japan) at an accelerating voltage of 10 kV. The surface morphology of the FBC samples was examined at a magnification of approximately 20,000×.

#### 2.5.3. Fourier-Transform Infrared (FTIR) Spectroscopy

For the attenuated total reflectance (ATR)–FTIR spectroscopic analysis (Perkin Elmer, Wellesley, MA, USA), the chemical structure of FBC was examined based on the generated spectrum within a spectral range of 4000–600 cm^−1^, with the signal obtained by averaging 30 scans at a resolution of 1 cm^−1^.

#### 2.5.4. Thermogravimetric Analysis (TGA)

The mass change of the FBC film was measured using TGA (TA Instruments Q50, New Castle, UK) to assess its thermal stability. FBC samples were analyzed under a nitrogen atmosphere with a flow rate of 40 mL/min. The temperature was increased from 25 to 600 °C at a heating rate of 10 °C/min.

### 2.6. Statistical Analysis

Statistical analysis of all experimental data (variation from baseline values) was performed using an analysis of variance (ANOVA). Post hoc comparisons with the negative control were carried out using Tukey’s test. All statistical analyses were conducted using IBM SPSS Statistics 19 (IBM, Armonk, NY, USA), with the significance level set to *p* < 0.05.

## 3. Results and Discussion

### 3.1. Production of Fungus-Immobilized FBC

**Figure 1:**
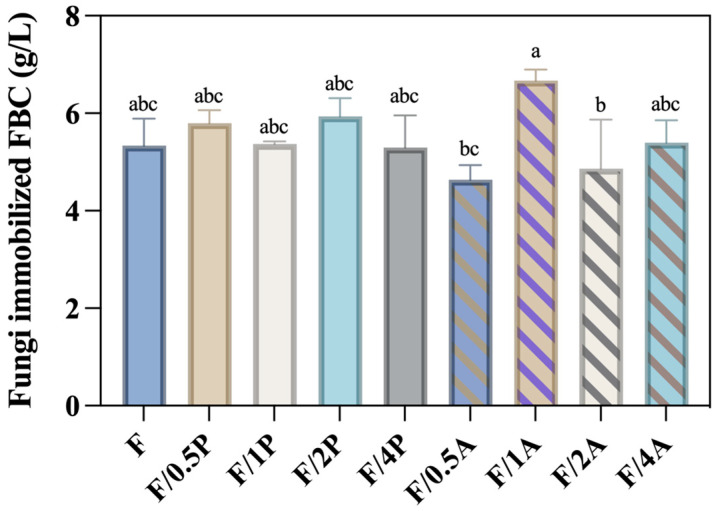
Production of *Pleurotus ostreatus* and *Aspergillus oryzae* immobilized on foamed bacterial cellulose (FBC). Different letters on error bars indicate significant differences (*p* < 0.05).

FBC is a composite fibrous biomaterial produced by microorganisms in a foamed culture medium. Due to its porous structure, it has been identified as a promising candidate for use as 3D cell scaffolds [24]. Furthermore, foaming reagents incorporated into the cellulose fibers during production render the FBC more resistant to cellulase degradation. As a result, FBC is more suitable as a support matrix for fungal microorganism cultivation compared to BC. Figure 1 shows no significant increase in weight with an increased ratio of fungal inoculum in either the *P. ostreatus*- or *A. oryzae*-immobilized FBC groups. The FBC/1A group exhibited the highest production (6.67 g/L), suggesting that this inoculation ratio achieved the most effective immobilization efficiency among all tested groups.

### 3.2. Water Content and Swelling Behavior of Fungus-Immobilized FBC

**Figure 2:**
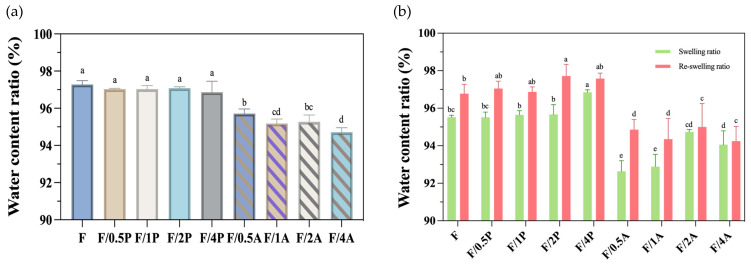
(**a**) Water contents and (**b**) swelling abilities of different fungus-immobilized foamed bacterial cellulose (FBC). Triplicate experimental results are expressed as the mean ± standard deviation. Different letters indicate significant differences from each other.

As shown in Figure 2a, the *P. ostreatus*-immobilized groups exhibited no difference in the original WC compared to the control group, with all values in the range of 96–97%. In contrast, the *A. oryzae*-immobilized groups showed a reduction in water retention, with values of around 94–95%. Nevertheless, both the *P. ostreatus*- and *A. oryzae*-immobilized groups maintained considerable WC levels.

In the swelling analysis presented in Figure 2b, the *P. ostreatus*-immobilized groups (inoculation ratios of 0.5P, 1P, 2P, and 4P) presented swelling rates in the range of 95.5–96.8%. After the first rehydration cycle, the reswelling ratio WC of the *P. ostreatus*-immobilized groups increased to a range of 96.8–97.7%. In contrast, those of the *A. oryzae* groups remained lower, with swelling ratios ranging from 92.6 to 94.7%, and increased reswelling behavior of 94.2–95%.

The rehydration results suggested that the increase in WC observed after the second rehydration may be attributed to structural loosening of the fibers. Under pressure of water absorption during rehydration, the pore size within the cellulose matrix may have expanded, and the inter-fibrillar spaces may have also increased, potentially leading to elevated WCs or a shift in the moisture content range. In addition, the structural integrity being compromised and fiber fragmentation occurring may also contribute to increased WCs upon rehydration. However, these hypotheses regarding changes in fiber structure require further validation through mechanical property analyses.

### 3.3. Morphological Analysis of Fungus-Immobilized FBC

**Figure 3:**
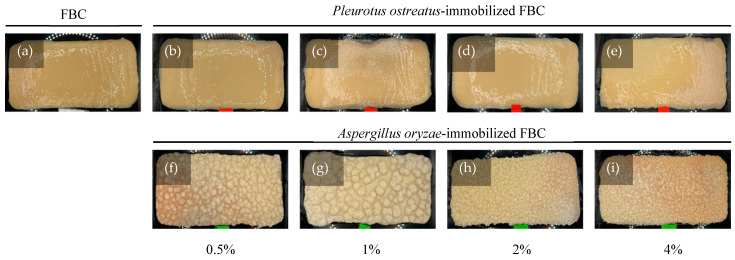
Appearance of fungus-immobilized foaming bacterial cellulose (FBC): (**a**) FBC, (**b**–**e**) *Pleurotus ostreatus*-immobilized FBC group, and (**f**–**i**) *Aspergillus oryzae*-immobilized FBC group.

**Figure 4:**
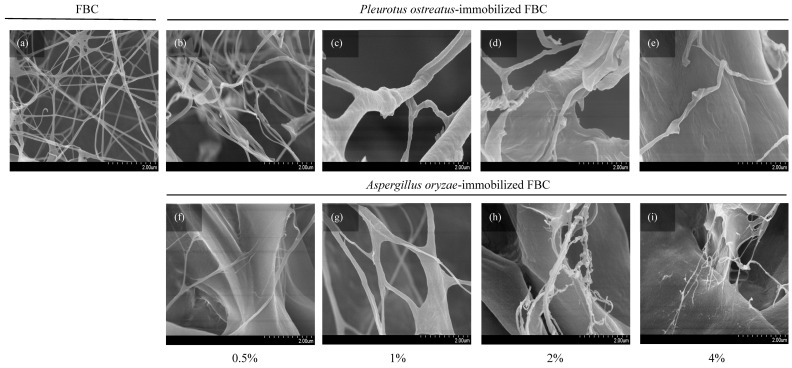
SEM images showing the surface morphology of foamed bacterial cellulose (FBC) scaffolds immobilized with different fungal species at various inoculation ratios: (**a**) FBC, (**b**–**e**) *Pleurotus ostreatus*-immobilized FBC group, and (**f**–**i**) *Aspergillus oryzae*-immobilized FBC group.

Fungal attachment to FBC used as a cultivation scaffold can be evaluated based on its surface morphology. The results (Figure 3a) revealed that FBC exhibited a smooth surface. In the *P. ostreatus* inoculation group (Figure 3b–e), the originally smooth surface became slightly roughened. In the *A. oryzae*-immobilized group (Figure 3f–i), a distinct rough and granular surface was observed. Furthermore, as the initial inoculation density increased, the sizes of the smaller particles of the granular morphology increased. The SEM results (Figure 4) showed that the network structures formed in the control group were similar to those reported in a previous study [25]. The *P. ostreatus*- and *A. oryzae*-immobilized groups exhibited significant morphological differences. In addition, both fungal species contributed to the formation of distinct fiber structures. In both the *P. ostreatus*- and *A. oryzae*-immobilized groups, in addition to the original cellulose fibers, fungal-derived fibrous structures were also observed, indicating the successful establishment of immobilization of fungi on FBC. Previous studies have demonstrated that fungal hyphae can penetrate deeply into the 3D network of BC nanofibrils during the co-culture process, thereby hindering the subsequent release of cells [26].

### 3.4. Characteristic Analysis of FBC

**Figure 5:**
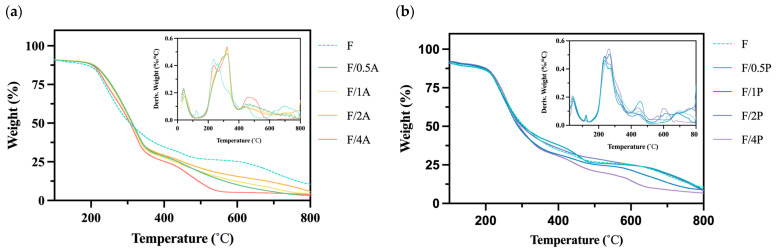
Results of a thermogravimetric analysis of different fungus-immobilized foaming bacterial cellulose (FBC): (**a**) *Pleurotus ostreatus*-immobilized FBC group, and (**b**) *Aspergillus oryzae*-immobilized FBC group.

BC is a biomaterial with high thermostability; therefore, an assessment of thermal stability is essential for characterizing BC-based biomaterials [27]. Figure 5 reveals a degradation rate curve in the range of 400–450 °C, which may be associated with breakdown of the fiber network structure. In previous studies, no significant changes in thermal stability were observed for FBC or the two fungal strains near 450 °C [14]. However, in the present analysis, a notable change was observed, potentially indicating that BC and fungal cells may have become entangled during immobilization to form a more thermally stable composite structure. This hypothesis warrants further investigation through a crystallinity analysis.

Moreover, as shown in Figure 5b, the group immobilized with *A. oryzae* exhibited a notable shift in the peak temperature of major thermal degradation. The peak shifted from approximately 220 to 320 °C, suggesting an enhancement in thermal stability under fungal immobilization. This shift may be attributed to the intrinsic thermal degradation characteristics of *A. oryzae*.

**Figure 6:**
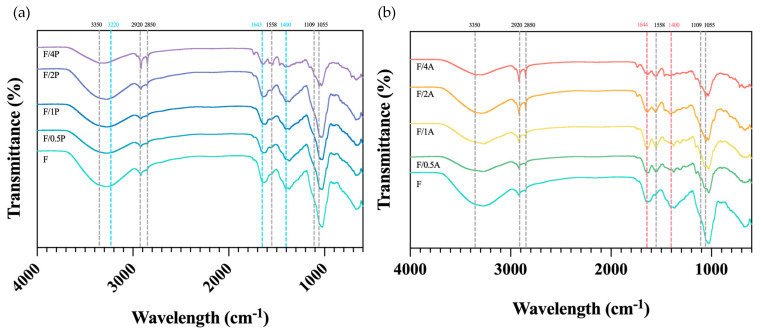
FTIR spectra of different fungus-immobilized foaming bacterial cellulose (FBC): (**a**) *Pleurotus ostreatus*-immobilized FBC group, and (**b**) *Aspergillus oryzae*-immobilized FBC group.

As shown in Figure 6a, the broad absorption band at 3350–3220 cm^−1^ corresponds to the O–H stretching vibrations of hydroxyl groups, while the bands at 2850–2920 cm^−1^ are attributed to C–H stretching vibrations of CH_2_ and CH_3_ groups. The absorption band at 1650 cm^−1^ was assigned to the H–O–H bending vibration of adsorbed water molecules in cellulose. The band at 1428 cm^−1^ represents CH_2_ bending vibrations, while the band at 1109 cm^−1^ corresponds to C–O stretching. The absorption at 1055 cm^−1^ is associated with C–O–C stretching vibrations related to functional groups in cellulose. These spectral features are in agreement with those previously reported for FBC [14].

According to previous studies [28], the FTIR spectrum of *P. ostreatus* exhibits distinct characteristic bands in the regions around 3320, 1643, 1571, 1400, and 1200–1000 cm^−1^. The band at 3320 cm^−1^ is likely due to N–H stretching vibrations of amide A in proteins and nucleic acids, or to O–H stretching from phenolic compounds and water. The 1641 cm^−1^ peak arises from C=O stretching in the amide I region, as well as C=C and C–O stretching and N–H bending vibrations associated with amino acids and flavonoids [29]. The 1571 cm^−1^ peak corresponds to amide II bands from N–H bending and C–N stretching in amino acids and proteins. The 1400 cm^−1^ band is attributed to symmetric stretching of COO^−^ groups in fatty acids and amino acids, symmetrical bending of methyl groups in skeletal proteins, and symmetrical stretching of methyl groups in proteins. The 1200–1000 cm^−1^ region is typically associated with C–O stretching vibrations in pyranose rings of carbohydrates. However, as shown in Figure 6, these characteristic fungal peaks are not clearly distinguishable. This may have been due to immobilization with FBC, which could have obscured fungal signals through overlap with dominant bacterial cellulose bands, or due to the relatively low fungal biomass, resulting in insufficient peak intensities for detection. When comparing the spectra of FBC and the FBC group immobilized with *A. oryzae*, a notable observation was the disappearance of the peak at around 1428 cm^−1^. This suggests that the presence of *A. oryzae* may contribute to the degradation or modification of this particular structural feature. However, the exact mechanism underlying this change requires further investigation and confirmation.

## 4. Conclusions

This study demonstrates the successful immobilization of *P. ostreatus* and *A. oryzae* on FBC scaffolds, which presents a promising avenue for various industrial applications. The thermal stability and water retention properties of these FBC composites underscore their robustness, making them suitable for diverse technological fields. Future studies could focus on fine-tuning the fungal concentrations to enhance specific performance attributes and exploring the potential of these composites in the development of sustainable and biodegradable materials. In addition, further investigations into the tunable mechanical properties of bacterial cellulose under varying relative humidity conditions, as well as its crystallinity and mechanical strength, could support future biotechnological innovations, such as microbial scaffolds for immobilization, functional packaging, and medical dressings.

## Data Availability

The original contributions presented in the study are included in the article, further inquiries can be directed to the corresponding author.

## Abbreviations

The following abbreviations are used in this manuscript

BC	Bacterial cellulose
FBC	Foamed bacterial cellulose
FTIR	Fourier-transform infrared spectroscopy
SEM	Scanning electron microscopy
TGA	Thermogravimetric analysis
